# Patient Satisfaction and Perception with Digital Complete Dentures Compared to Conventional Complete Dentures—A Pilot Study

**DOI:** 10.3390/dj13070291

**Published:** 2025-06-27

**Authors:** Andrea Bors, Melinda Szekely, Liana Beresescu, Alexandra Maier, Felicia Beresescu

**Affiliations:** 1 Faculty of Dentistry, George Emil Palade University of Medicine, Pharmacy, Science, and Technology; 38 Gheorghe Marinescu Street, 540142 Targu Mures, Romania; 2 ISmileLab, Bd 1848 Street, 540429 Targu Mures, Romania

**Keywords:** additive manufacturing, CAD/CAM, complete denture, dental materials, denture teeth, digital dentures, digital impression

## Abstract

**Background:** Patient satisfaction is a critical outcome in the rehabilitation of edentulous patients. While conventional fabrication methods are widely used, digital workflows are emerging as viable alternatives. However, direct comparative evidence from the patient’s perspective remains limited. **Objective:** To compare patient satisfaction between conventional complete dentures (C-CD) and digital complete dentures (D-CD) in maxillary edentulous patients, including changes in perceptions over time and final prosthesis preference. **Methods:** A prospective, randomized crossover clinical trial was conducted in 2023–2024 involving 40 completely maxillary edentulous patients meeting specific inclusion criteria. Participants were randomly allocated into two sequence groups: Group 1 (n = 20) received C-CD first, and Group 2 (n = 20) received D-CD first, each for 6 months (T1), followed by crossover to the alternate denture for another 6 months (T2). Patient satisfaction was measured using a 10-item questionnaire at 6 and 12 months. **Statistical analysis:** Wilcoxon signed-rank tests were used for within-subject comparisons of denture types, and Mann–Whitney U tests for between-group comparisons, with significance set at *p* ≤ 0.05. **Results:** Using the paired crossover analysis, D-CD showed significantly better comfort than C-CD (*p* < 0.05). D-CD scored significantly higher than C-CD in most satisfaction domains, including comfort, retention, speech, esthetics, and need for adjustments (*p* ≤ 0.05). Median scores for retention, speech, esthetics, and other domains were slightly higher with D-CD but did not reach statistical significance (*p* > 0.05). Additionally, the D-CD required fewer post-insertion adjustment visits than the C-CD (*p* < 0.05). By the end of the trial, 28 patients (70%) preferred the digital denture as their final prosthesis, whereas 12 patients (30%) preferred the conventional denture. **Conclusions:** Incorporating digital technology in the fabrication of complete dentures significantly enhances patient satisfaction compared to conventional methods. This study highlights the clinical relevance of modern dental prosthesis technology and supports the wider integration of digital workflows. Within the limitations of this pilot study, digitally fabricated complete dentures provided overall patient satisfaction comparable to conventional dentures, with the D-CD offering a notable improvement in comfort. The majority of patients ultimately favored the digital denture, supporting the clinical viability of CAD/CAM workflows.

## 1. Introduction

Complete edentulism continues to pose a significant public health concern, particularly among aging populations, with reported prevalence ranging between 7% and 70% globally, depending on the age group and region [1,2,3]. In Romania and other parts of Eastern Europe, edentulism remains relatively high due to delayed access to oral healthcare and limited prosthodontic interventions in underserved communities [4,5].

For decades, conventional complete dentures (C-CD) have been the primary approach to fully rehabilitating edentulous patients. These prostheses, fabricated through traditional analog workflows, require multiple clinical steps including impressions (preliminary and final), bite registrations, try-ins, and adjustments, along with the laboratory stages: pouring preliminary and master cast, manufacturing a custom tray, wax rim and record base, wax up, and processing the denture. While time-tested, conventional dentures are often associated with clinical limitations, such as material shrinkage, tissue distortion, reduced retention, and the need for frequent post-insertion corrections [6,7].

Recent advancements in dental technology have introduced digitally fabricated complete dentures (D-CD) using computer-aided design and computer-aided manufacturing (CAD/CAM), milling, or 3D-printing techniques. These approaches offer significant benefits, including improved precision and reproducibility, enhanced tissue adaptation, faster technological timelines, and better control over occlusion and aesthetics [8,9]. Digital workflows also reduce the number of clinical appointments, which is particularly beneficial for elderly patients with limited mobility or systemic conditions [10].

Patient satisfaction and comfort are pivotal outcomes in complete denture therapy. Edentulous patients rely on well-fitting dentures for function and quality of life, so prosthesis acceptability is critical. Conventional complete denture fabrication methods have been successfully used for decades, but in recent years, digital workflows (computer-aided design and manufacturing of dentures) have emerged as a modern alternative. These digital techniques promise reduced clinic visits and potentially improved fit due to computerized precision. However, the impact of digital versus traditional denture fabrication on patient satisfaction remains uncertain [11]. The existing literature provides limited and sometimes conflicting evidence from the patient’s perspective. For instance, one randomized crossover trial [11] reported that patients with digitally fabricated (3D-printed) dentures experienced lower satisfaction in certain domains (such as stability and comfort) compared to those with conventional dentures. In contrast, another study [12] found no significant difference in patient-reported oral health quality of life between digital and conventional complete dentures. Such mixed findings highlight the need for further comparative research focusing specifically on patient satisfaction and perception.

Given the rapid adoption of digital denture technology [13] and the importance of patient-centered outcomes, there is a strong justification for this investigation. Our study was designed as a pilot to generate preliminary data on patient satisfaction with digital complete dentures compared to conventional dentures. We aimed to determine whether digitally fabricated dentures could meet or exceed the satisfaction levels of conventional dentures in a controlled clinical setting. This study further aimed to analyze intra-individual variations in patient-reported outcomes associated with sequential exposure to both denture types and to determine the definitive prosthetic preference based on cumulative user experience. By using a crossover design, each patient served as their own control, which we anticipated would provide a sensitive comparison between the two fabrication methods. The null hypothesis was that there would be no difference in patient satisfaction between digital and conventional complete dentures.

Our research was performed to bridge the gap by allowing patients to directly experience both prosthetic options before expressing a preference. The primary aim of this study was to compare patient satisfaction with conventional and digital complete maxillary dentures in a fully edentulous population using a prospective, randomized, crossover design.

Secondary objectives included the following:

- Assessing the change in patient perception after experiencing both prosthesis types.
- Evaluating the internal consistency of a patient satisfaction questionnaire (PSQ).
- Identifying the preferred prosthesis at the end of the study and patient recommendations for future users.

By integrating validated tools and allowing patients to evaluate both treatment modalities, this study aims to provide a robust, patient-centered comparison that reflects real-world clinical decision-making.

## 2. Materials and Methods

### 2.1. Participants

Forty complete maxillary edentulous patients were selected between 2023 and 2024 from the Faculty of Dentistry at George Emil Palade University and affiliated private practices in Târgu Mureș, Romania, based on the following inclusion criteria: patients with complete maxillary edentulism, good oral hygiene, free of any systemic diseases that may affect oral health, and no contraindications to the materials used for complete dentures. The patients included in this study had varying conditions in the opposing mandibular arch, including complete dentures (45%), removable partial dentures (35%), or fixed prosthetic restorations (20%). The presence of different mandibular arch conditions was considered when analyzing the results, as it may influence satisfaction with the maxillary dentures. The exclusion criteria were uncontrolled systemic disease, infectious diseases, partial maxillary edentulism, temporo-mandibular disorders, xerostomia, oro-facial pain, patients who could not read the informed consent form, patients with congenital or acquired defects in the maxilla and/or mandible, and patients considered ineligible for study inclusion by the principal investigator.

All participants signed an informed consent form that included a description of the intervention.

### 2.2. Study Design

**Figure 1:**
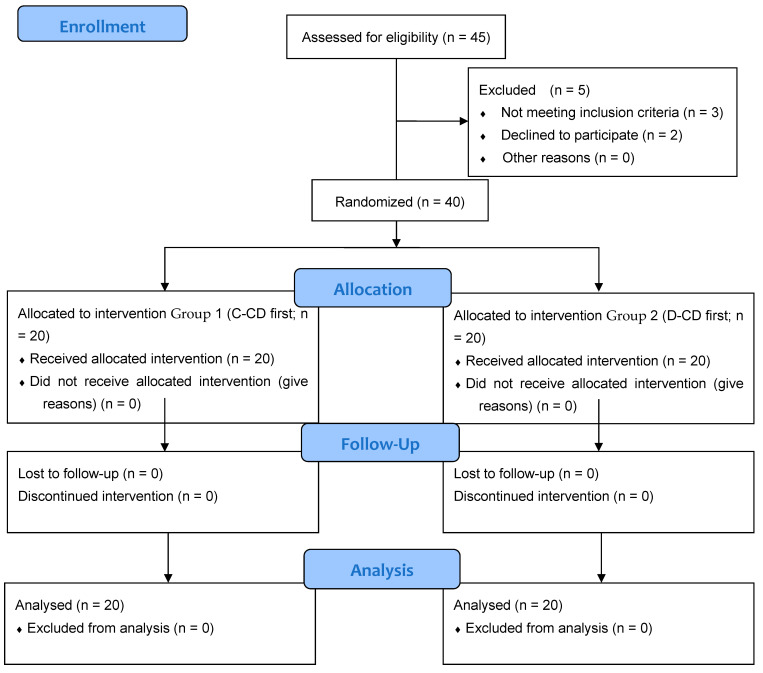
CONSORT 2010 Flow Diagram.

The trial was designed as a prospective, randomized, single-center study. The study protocol was approved by the Ethics Committee of Dentexpert SRL (Approval No: 1792/20.01.2023; Approval Date: 20 January 2023). No deviations were made from the registered protocol. The clinical trial was conducted in accordance with the Declaration of Helsinki and Consolidated Standards of Reporting Trials (CONSORT) guidelines. The trial was registered in a public ISRCTN registry with the number ISRCTN47410. The flow of participants throughout the study is illustrated in Figure 1.

The two prosthetic restorations assessed were the maxillary conventional complete denture (C-CD) and digital maxillary complete denture (D-CD). 

Randomization and Crossover Procedure: Participants were randomly allocated into two equal groups to determine the order of denture type received. A computer-generated random sequence was used for allocation, and group assignments were concealed in sealed opaque envelopes opened after each patient’s enrollment. Group 1 (n = 20) received a conventionally fabricated complete denture (C-CD, (Ivobase + Phonare II, Ivoclar, Schaan, Liechtenstein)) first, followed by a digitally fabricated complete denture (D-CD, CAD/CAM-designed, Denture 3D+, NextDent NextDent B.V., (Soesterberg, The Netherlands) + Harz Labs Dental Sand (Harz Labs LLC, Moscow, Russia), while Group 2 (n = 20) received the D-CD first, then the C-CD. Each patient used the first denture for a period of 6 months (T1), after which they crossed over to the alternate denture type, which was then used for another 6 months (T2). There was no washout period between treatments, as removing a functional denture for an extended time was not feasible; however, a 1–2 week adaptation period was allowed with the new prosthesis before data collection at each crossover point. Importantly, each patient received a new maxillary fabricated by both methods. This means that during each phase, the patient wore a conventional or digital denture. All patients received thorough instructions on denture use and maintenance at delivery.

Conventional Denture Fabrication: Conventional complete dentures were fabricated following standard clinical and laboratory protocols. First, preliminary impressions of the edentulous ridges were made using alginate in stock trays. From these, preliminary casts were obtained, and custom trays were fabricated. The borders of the custom tray were adjusted, and border molding was performed using modeling compound to achieve proper extension. Final impressions were taken with medium-body polyvinyl siloxane (PVS) material to capture detailed anatomy. Master casts were poured in dental stone from the final impressions. On each master cast, a baseplate and wax rim were constructed; these record bases were used to record the maxillomandibular relationship (jaw relations) and to determine vertical dimension and centric relation. The tooth selection (shape and shade) was performed per the patient’s esthetic preferences and the prosthodontist’s guidance. The teeth (acrylic denture teeth) were arranged in wax on the master casts, and a wax try-in was conducted for each patient. After verification of fit, occlusion, and esthetics at the try-in stage, the dentures were processed in heat-cured polymethyl methacrylate resin using the compression molding technique. The finished conventional dentures were then deflasked, trimmed, and polished. Any necessary laboratory remount and occlusal adjustments were performed to refine occlusion. All laboratory procedures were carried out by the same experienced dental technician to ensure consistency in denture fabrication quality.

Digital Denture Fabrication: Digital Impressions and Jaw Relations: For patients in the digital denture group, a complete intraoral scanning workflow was employed. Edentulous maxillary and mandibular arches were captured using a high-resolution intraoral scanner (Medit i700, Medit Corp., Seoul, South Korea) to obtain precise digital impressions of the tissue surfaces. To record maxillomandibular relations (vertical dimension of occlusion and centric relation), conventional wax occlusion rims were used on 3D-printed custom record bases. In each case, the wax rim assembly (with the established occlusal vertical dimension and centric bite) was either scanned in the patient’s mouth or extra-orally to digitize the jaw relation record. In some cases, an intraoral gothic-arch tracing device was additionally used to fine-tune centric relation; the tracer markings or plates were likewise scanned and aligned with the arch scans. This ensured that the patient’s bite registration was accurately transferred into the virtual environment. All digital impressions and bite records were made by the same clinician to maintain consistency across cases.

Virtual Denture Design (CAD): The digital arch models and jaw relation records were imported into a specialized dental CAD software for complete dentures (exocad DentalCAD Matera 2.4, GmbH, Germany). Using this software, a virtual denture setup was designed for each patient. The anatomical landmarks (e.g., midline, smile line, and occlusal plane) guided the placement of artificial teeth from the software’s tooth library. The maxillary dentures were designed in proper occlusion according to the recorded centric relation and vertical dimension. A virtual articulator function was used to simulate mandibular movements, allowing adjustment of tooth positions to achieve balanced occlusion in excursions. The denture bases were contoured in the software to optimal form and extension, and relief areas were incorporated as needed. The final approved denture design consisted of two sets of STL files—one for the denture base (with sockets for teeth) and one for the denture teeth—exported for 3D printing.

Three-Dimensional Printing and Post-Processing: The complete dentures were fabricated by additive manufacturing using a stereolithography-based 3D printer. In this study, a digital light processing (DLP, Asiga, Alexandria, Australia) printer was used to achieve high accuracy and resolution (layer thickness ~50 μm) for the denture components. Each denture base was printed in a pink biocompatible denture resin (NextDent Denture 3D+, Vertex-Dental B.V., Soesterberg, Netherlands), while the teeth were printed separately in a tooth-colored microfilled hybrid resin (Harz Labs Dental Sand, shade A3, Harz Labs, Riga, Latvia). The NextDent Denture 3D+ material is a Class IIa medically certified resin with mechanical properties comparable to conventional heat-cured PMMA denture base materials (flexural strength ~84 MPa; flexural modulus ~2380 MPa) [14]. The Harz Labs Dental Sand resin is a methacrylate-based composite designed for dental applications, characterized by high hardness (~90 Shore D) and strength, making it suitable for durable denture teeth [15]. Printed parts were cleaned of residual resin by rinsing in isopropyl alcohol baths and then post-cured in a light-curing unit (manufacturer-recommended UV oven) to ensure complete polymerization. Any support structures were carefully removed, and the denture base and teeth components were finished as per standard protocols. For assembly, the printed teeth were bonded into the corresponding sockets of the printed base using a light-cured bonding resin matching the denture base material. The assembled dentures were then polished to a smooth finish, especially along the borders and occlusal surfaces. Prior to delivery, each digital denture underwent occlusal adjustment on an articulator (or intraorally) to eliminate any prematurities and to refine centric contacts and balancing contacts. The entire fabrication process for all digital cases was carried out by the same prosthodontist-technician team, standardizing the clinical and laboratory techniques and thereby ensuring consistency across the digital denture cohort.

### 2.3. Data Collection

I. Baseline Data: Age, gender, dental history, reason for tooth loss.

II. Patient Satisfaction Questionnaire (PSQ) (Table 1), adapted for removable complete dentures [15]. During the follow-up of the two periods, patients were asked to complete a patient satisfaction questionnaire after 6 (T1) and 12 (T2) months with each denture. Patient satisfaction and perception were evaluated using a structured 10-item questionnaire covering various domains of denture experience. The questionnaire was administered after 6 months with each denture. Each item was scored on a Likert scale from 1 to 5 (1 = very dissatisfied, 5 = very satisfied) for all participants. The domains assessed included comfort, retention (security of fit), stability during function, ease of speaking (speech), ability to chew food, aesthetics (appearance), ease of cleaning the denture, need for adjustments (perceived need for post-delivery corrections), confidence in social situations, and overall satisfaction. These domains were chosen to encompass both functional and psychological aspects of denture acceptance. Patients completed the questionnaire independently, and the researcher verified completeness but did not influence responses. Higher scores indicated better satisfaction/perception in each domain. The primary outcome of interest was the difference in satisfaction scores between the D-CD and C-CD for each domain, with particular attention to whether one type of denture led to higher comfort or overall satisfaction. The secondary outcomes included the total number of adjustment visits required for each denture type and the patient’s final preference for denture.

Patients were scheduled for follow-up visits at 2 weeks and 1 month after insertion of each denture to address any sore spots or perform minor adjustments. The number of post-insertion adjustment appointments required for each denture was recorded for each patient. After 6 months of using the first set of dentures, patients returned for evaluation before crossover. At this 6-month visit (T1), data on patient satisfaction were collected (as described below). Patients then switched to the alternate set of dentures (either receiving the new digital set if they had conventional first, or vice versa). After another adaptation period and any needed initial adjustments, a similar follow-up schedule was maintained. At 12 months (T2), the final evaluation was performed. At this final visit, patient satisfaction was assessed again, and patients were asked to indicate which denture (digital or conventional) they preferred overall and wished to keep using going forward. Because this was a pilot study, all patients were ultimately provided with the denture set of their choice (either they kept the digital or the conventional set based on preference, and if they preferred the digital, the conventional set was kept as a spare, or vice versa).

Timeline

- Baseline (day of delivery).
- Six (T1) and twelve (T2) month follow-up.

### 2.4. Data Analysis

All data were analyzed using SPSS (v.25, IBM Corp) on an intention-to-treat basis (all enrolled patients completed the crossover). We first performed descriptive statistics for all variables. Given the crossover design, each patient provided paired data (satisfaction scores with C-CD vs. the same patient’s scores with D-CD). Data normality was checked using the Shapiro–Wilk test, which indicated that the satisfaction scores were not normally distributed in several domains. Therefore, we chose non-parametric tests for analysis. For the primary comparisons of satisfaction outcomes between denture types, we used the Wilcoxon signed-rank test (a non-parametric paired test) to compare median domain scores for D-CD versus C-CD within the same patients. This test effectively evaluates whether there is a systematic difference in satisfaction when patients switch from one denture to the other. Additionally, to ensure that any potential group or period effects did not confound results, we conducted a preliminary check: the two groups (with different sequence orders) were compared at the 6-month mark (after the first period) using the Mann–Whitney U test for each domain to see if starting denture type had an initial influence. We also compared the magnitude of change in scores after crossover between groups to assess carryover. No significant sequence or carryover effects were detected (*p* > 0.05 for all such comparisons), justifying pooling the crossover data for within-subject analysis. For the number of adjustment appointments, we used a Wilcoxon matched-pairs comparison as well. The final denture preference (digital vs. conventional) was summarized descriptively and analyzed by a binomial test to see if one was chosen significantly more often than the other. A two-tailed *p*-value ≤ 0.05 was considered statistically significant for all tests.

## 3. Results

### 3.1. Baseline Characteristics

All 40 enrolled patients completed both phases of the study and were included in the analysis. There were no dropouts. The mean age of participants was 65.3 ± 8.7 years, with 16 females and 24 males (Table 2). At baseline, all participants were existing denture wearers (having worn their previous conventional dentures for at least one year) and expressed a desire for improved dentures. The two randomized sequence groups (conventional-first vs. digital-first) were similar in terms of demographics and oral characteristics; there were no significant differences in age, gender distribution, or ridge anatomy scores between the groups.

### 3.2. Patient Satisfaction Questionnaire (PSQ)

All patients successfully adapted to both types of new dentures over the course of the trial. When comparing the digital complete dentures (D-CD) to the conventional complete dentures (C-CD), we found that overall patient satisfaction was comparable between the two fabrication methods in most respects, with one notable exception. Specifically, patient comfort while wearing the dentures was significantly better with the digital dentures. On a 1–5 scale, the median comfort rating was 5 (very satisfied) for D-CD and 4 for C-CD; this difference was statistically significant (*p* = 0.02, Wilcoxon signed-rank test). For other domains of satisfaction—including retention (how well the denture stayed in place), stability during chewing, ease of speaking, esthetics, ease of cleaning, and overall satisfaction—there were no statistically significant differences between D-CD and C-CD. In these categories, patients’ ratings for the digital dentures were on par with, and in some cases slightly higher than, their ratings for the conventional dentures, but the differences did not reach our significance threshold. For example, median retention satisfaction was 5 for D-CD vs. 5 for C-CD in most cases (*p* = 0.08), median speech satisfaction was 4 vs. 4 (*p* = 0.15), and overall satisfaction was 5 vs. 5 (*p* = 0.33). These *p*-values indicate trends but no reliable differences in those aspects. Thus, aside from comfort, patients perceived both types of dentures as similarly satisfactory in terms of function and appearance after a 6-month adaptation to each.

Table 3 presents the results for all PSQ domains across both evaluation points. At the 6-month follow-up (T1), digital complete dentures (D-CD) demonstrated significantly higher patient satisfaction compared to conventional complete dentures (C-CD) in the domains of comfort (*p* = 0.04), retention (*p* = 0.04), mastication (*p* = 0.04), and need for adjustments (*p* = 0.02). No significant differences were observed in aesthetics, stability, speech, oral condition, or overall satisfaction at this point.

At the 12-month follow-up (T2), after the crossover period, patient satisfaction with D-CD was significantly higher than with C-CD across almost all domains, including comfort (*p* < 0.01), aesthetics (*p* = 0.03), stability (*p* < 0.01), speech (*p* < 0.01), retention (*p* < 0.01), mastication (*p* < 0.01), need for adjustments (*p* < 0.01), oral condition (*p* = 0.03), and overall satisfaction (*p* < 0.01), (Table 3).

### 3.3. Internal Consistency

High internal consistency was observed across all PSQ data sets, with Cronbach’s alpha coefficients exceeding 0.90 (Table 4). This indicates excellent reliability of the questionnaire responses and supports the credibility of patient-reported satisfaction.

### 3.4. Change in Patient Perception

In terms of clinical performance, one practical difference observed was in the number of post-delivery adjustment visits required. Digital dentures require fewer adjustment appointments on average than conventional dentures. For the D-CD, the median number of follow-up adjustment sessions (after initial delivery) was 1, whereas for the C-CD it was 2. Twenty-nine patients (72.5%) needed no more than one minor adjustment with the digital denture, while with the conventional denture, thirty patients (75%) required two or more adjustments for sore spots or occlusal discrepancies. This difference was statistically significant (*p* = 0.01 on a paired comparison of adjustment counts). This suggests that the fit of the digital dentures, as delivered, may have been more accurate, resulting in fewer pressure spots or occlusal corrections. However, by the 6-month evaluation, all dentures (of both types) were adequately adjusted and comfortable, as reflected in the high satisfaction scores mentioned above.

We assessed whether the order in which patients received the dentures influenced their experience (a potential period or carryover effect). Importantly, no significant period effect was found. Patients who started with digital and then switched to conventional reported similar satisfaction with the conventional denture as those who started with conventional first (and vice versa). There was no evidence of persistent adaptation from the first denture affecting the second; the satisfaction ratings for each denture type were consistent regardless of sequence (*p* > 0.1 for comparisons between sequence groups at each phase). This lack of carryover effect validates our approach of analyzing the data as paired within-subject comparisons. We did observe that some patients subjectively commented that adjusting to the second denture was easier than the first simply because they “knew what to expect,” but this did not translate into any measurable difference in the satisfaction scores attributable to sequence. Overall, the crossover design was effective, and each patient’s comparison between the two denture types can be considered independent of the sequence.

### 3.5. Patient Preference and Recommendations

After experiencing both types of dentures, patients were asked at the end of the study which denture they preferred and intended to continue using. The majority of participants (28 out of 40, or 70%) expressed a preference for the digital complete denture as their final prosthesis. The remaining 12 patients (30%) preferred the conventional denture. By the end of the study, 28 patients (70%) chose to keep using the D-CD, whereas 12 patients (30%) favored the C-CD. This indicates a substantial tilt toward the digital option, aligning with the comfort advantage noted earlier. Many of those who preferred the D-CD cited its superior comfort and fit as the primary reason. Some also mentioned subjective reasons such as “felt more natural” or “liked the look” of the digital denture, although objectively, esthetic scores were similar. Among the 12 patients who preferred the conventional denture, a common reason was that they had become very accustomed to it in the first 6 months and felt it was “already broken in,” whereas a few others believed the conventional denture felt slightly more stable during heavy chewing for them. It is worth noting that all 12 of those patients still rated their digital denture experience as satisfactory; their choice often came down to personal habit or subtle preference. In summary, while satisfaction metrics were equivalent in most domains, when forced to choose one denture, most patients opted for the digital, likely influenced by its comfort and possibly fewer visits for adjustments (convenience).

## 4. Discussion

This randomized crossover pilot study compared patient perceptions of digitally fabricated versus conventionally fabricated complete dentures. Our key finding was that patient-reported satisfaction was largely equivalent between the two types of dentures, with the exception of comfort, which was significantly improved in the digital dentures. In practical terms, patients found both their conventional and digital dentures acceptable in terms of retention, stability, esthetics, speech, and overall satisfaction after an adequate adjustment period. However, they reported higher comfort with the digital dentures, and this comfort advantage may have contributed to the majority’s preference for the digital prosthesis at the trial’s conclusion.

Comfort is a critical aspect of denture success, as discomfort can deter patients from wearing their dentures consistently. The improved comfort with digital dentures observed in our study could be attributed to the precision of the digital fabrication process. The CAD/CAM 3D printed dentures were created from accurate digital models, potentially resulting in a better initial fit to the patient’s tissue surfaces. Indeed, our data on post-insertion adjustments support this: the digital dentures required fewer adjustments for sore spots, implying a more uniform tissue adaptation from the outset. Less adjustment translates not only to comfort but also to reduced chair time and inconvenience for the patient, which are important factors in overall satisfaction. This finding is clinically significant even though other domains did not differ; comfort alone can be a deciding factor in a patient’s preference, as seen by those who chose the digital denture.

It is noteworthy that aside from comfort, at T1, we did not find significant differences in other domains of satisfaction. This suggests that when both types of dentures are made carefully and fitted well, their functional outcomes (such as stability during chewing or clarity of speech) can be comparable. This aligns with the results of Zupancić et al. [12], who also reported no major differences in patient-reported outcomes (using OHIP-20 scores) between digital and conventional dentures in a controlled trial. Our study reinforces the idea that the overall efficacy of digital dentures is on par with the traditional approach for most patient-centered measures.

Our findings, however, differ in part from those of Katsura et al., who found in 2022 that conventional dentures had higher patient satisfaction in several domains compared to 3D-printed digital dentures [11]. In Katsura’s crossover study, conventional dentures were favored in aspects like stability, phonetics, and general satisfaction, whereas only a minority of patients preferred the digital option. The discrepancy between Katsura’s results and ours could be due to differences in the digital fabrication method and materials. Their study utilized 3D-printed dentures, which might have differences in surface fit or material properties (e.g., the resin used for printing might not adapt to tissue as well or could be less comfortable). Additionally, our patient population and inclusion criteria differed; all our patients received dentures, whereas in some other studies, patients might have used old dentures during one phase or had varying prior experiences. Another factor is the learning curve and technique; our operators and technicians were experienced in digital processes, and we attempted to standardize clinical steps between the two methods as much as possible. As digital denture technology and techniques improve, outcomes may become more consistent.

Another interesting outcome in our study was the final denture preference. Even though objective scores for most domains were similar, a significant majority of patients chose the digital denture when asked to pick one. This subjective preference underscores the importance of subtle factors that might not all be captured by quantitative scores. Patients often consider the sum of their experience: comfort (as discussed) and also possibly the convenience of fewer adjustments, or even intangible perceptions that the digital denture was “more modern” or required less effort to become used to. Psychologically, knowing they were using a state-of-the-art product may have positively influenced some patients’ perceptions—a form of technological confidence. We tried to mitigate bias by not explicitly labeling dentures as “better” or “high-tech” to the patient, but we cannot rule out a placebo effect of new technology. That being said, given that many had tangible comfort benefits, their preference is well-founded. For the minority who preferred the conventional denture, it highlights that individual variation plays a role; some might simply have found their conventionally made denture to be equally good and stuck with what was familiar.

Clinical Implications: The results of this pilot study suggest that digital complete dentures are a viable alternative to conventional dentures from the patient’s perspective. A significant comfort improvement with digital dentures can enhance patient experience, and at the very least, digital methods did not compromise any aspect of satisfaction. For clinicians, this means that adopting digital denture workflows can be performed with the confidence that patient outcomes (in terms of satisfaction) will be on par with traditional techniques. Additionally, the reduction in adjustment visits for digital dentures can save clinical time and improve efficiency. This benefit is advantageous for busy practices and for patients who have difficulty returning for multiple appointments. The crossover nature of the study also provided a unique opportunity for patients to effectively test both types of dentures on themselves; many were amazed at being able to compare and were ultimately happy to have a denture that they felt was the best for them.

Limitations: We acknowledge that this study has limitations. Firstly, as a pilot study with 40 patients, the sample size is relatively small. While it was sufficient to detect a difference in comfort, the study may have been underpowered to detect smaller differences in other domains of satisfaction. For example, some domains showed trends favoring the digital dentures (e.g., retention had *p* ~0.08); a larger sample might clarify whether these trends could reach significance or confirm they are truly equivalent. Secondly, our follow-up duration was limited to 12 months. Long-term differences in prosthesis durability, patient satisfaction over years of use, or maintenance issues (such as tooth wear or base relining needs) were not captured. It would be valuable to follow these patients longer-term. Thirdly, although we attempted to minimize bias, patients and clinicians were not blinded to the type of denture (it is inherently obvious due to differences in fabrication process and appearance of the workflow). This lack of blinding could introduce some bias in patient-reported outcomes. However, the crossover design mitigates this to some extent, as each patient compared both types. Finally, our digital fabrication method (printing) is only one of several available; results might differ with milled dentures or other digital systems.

We did not formally incorporate an objective measure of chewing efficiency or functional analysis in this pilot (besides patient perception), which could be included in future studies to correlate with satisfaction. Additionally, while we checked for carryover effects and found none significant, it is possible that a longer “washout” or a different sequence might reveal subtle adaptation phenomena; however, ethically and practically, a washout without dentures wasn’t possible in our design.

Future Directions: The encouraging results of this pilot support conducting a larger randomized trial. Future studies with a greater number of participants can further validate the comfort benefit and evaluate if other domains might show differences with improved power. It would also be useful to explore quality of life measures (e.g., OHIP-EDENT) and performance metrics (like bite force or chewing efficiency tests) alongside satisfaction ratings to provide a comprehensive comparison. Additionally, cost-effectiveness and time efficiency analyses could be integrated, as digital dentures may reduce clinical time but involve different laboratory costs; these factors ultimately also impact patient satisfaction (in terms of convenience and possibly cost satisfaction). We also plan to investigate the long-term maintenance, for example, how each type of denture fares in terms of the need for relines or tooth fracture over several years. Patients’ adaptation process could be studied more closely too; some anecdotal feedback suggested the second denture was easier to become used to, regardless of type, which is an interesting psychological aspect that could be quantified in future research (perhaps by measuring patient confidence or learning effect).

The CAD/CAM and 3D printing technologies used in digital denture fabrication improve base adaptation [16] and reduce processing errors such as polymerization shrinkage or porosity [17,18]. Digital dentures often provide better mucosal contact and retention due to accurate scanning and virtual design, enhancing stability and reducing sore spots [19]. This was reflected in the significant improvement in retention, comfort, and adjustment-related satisfaction scores in our study.

Patient satisfaction is not solely functional; it also encompasses aesthetic perception, psychological comfort, and social reintegration. Patients in our study favored D-CD in esthetics and speech clarity after the crossover, likely due to improved tooth positioning and phonetic design facilitated by digital articulation software. Similar findings were documented by Bidra et al. [20], who found that digitally designed tooth arrangements enhanced smile lines and lip support. Moreover, psychological literature highlights that empowering patients with modern, tech-based solutions can enhance confidence and perception of care quality [21]. As digital dentures are often associated with innovation and progress, patients may perceive them as higher quality, even prior to the wear experience.

The crossover design uniquely enabled the observation of patient perception changes, a valuable insight often overlooked in parallel-group studies. Initially, at T1, the differences between C-CD and D-CD were moderate. However, after experiencing both prostheses, patients clearly favored the digital dentures at T2. This evolution suggests that initial familiarity with conventional prosthetics may bias early satisfaction scores, but actual comparative experience reveals the superiority of digital solutions.

As noted by Zandinejad et al. [13], some patients initially hesitate to adopt digital dentures, but adaptation and perception improve over time, especially when the digital prosthesis is better customized to the individual’s anatomy.

Digital workflows significantly reduce clinical chair time and visits, which is particularly beneficial for elderly or systemically compromised patients. Several studies report time savings of 30–50% with D-CDs compared to traditional methods [19]. This advantage, coupled with fewer post-insertion visits (as seen in our low adjustment needs), makes D-CDs a viable solution in both public and private dental systems.

In settings like Romania, where dental care access is uneven, streamlined digital workflows may help reduce treatment barriers and standardize prosthetic outcomes in underserved communities [4,21]. This echoes findings by Singh et al. [22], who reported that patient satisfaction with digital dentures increased notably after a two-week adaptation phase. It suggests that initial resistance to digital prostheses is often temporary and resolves with wear experience.

The high internal consistency (Cronbach’s α > 0.90) across all questionnaires and scales adds robustness to our findings and confirms the reliability of patient feedback. The inclusion of a validated 10-item satisfaction questionnaire provided comprehensive coverage of patient-reported outcomes. Similar reliability has been observed in prior prosthodontic surveys using multi-item tools [23], reinforcing the value of using standardized, patient-reported outcome measures (PROMs) in clinical research.

Moreover, by involving patients with various mandibular opposing arch conditions, this study reflects a real-world edentulous population, increasing the external validity of the findings.

This design revealed a significant change in perception, with most patients favoring the digital option after having experienced both. Initially, some domains showed minimal differences, likely due to familiarity bias toward conventional prosthetics. However, by the end of the trial, the majority of participants (60%) preferred D-CD, and this preference was more pronounced in males.

The consistent preference for D-CDs suggests several clinical benefits:

- Fewer follow-up visits due to reduced need for adjustments.
- Improved patient compliance and adaptation, particularly for first-time denture wearers.
- More efficient fabrication processes, reducing chair time and lab coordination.

As digital workflows become more accessible and affordable, these advantages could significantly enhance the standard of care in prosthodontics, especially for underserved populations.

Despite its strengths, the study has several limitations. The lack of a wash-out period could have introduced residual adaptation bias. However, this was ethically justified, as prolonged maxillary edentulism is not clinically acceptable. Additionally, long-term follow-up was beyond the scope of this study. Future research should examine the durability, maintenance, and long-term satisfaction of digital dentures over periods exceeding one year.

Also, the one-year follow-up period may not capture long-term wearability or prosthesis durability. Future studies should explore long-term satisfaction and maintenance over 12–24 months. However, a recent systematic review and meta-analysis revealed that milled complete dentures would be recommended in dental practice [23].

## 5. Conclusions

In summary, this pilot crossover study indicates that digitally fabricated complete dentures can achieve patient satisfaction outcomes comparable to those of conventionally fabricated dentures. Comfort was significantly improved with the digital dentures, which is a meaningful advantage from the patient’s standpoint. Consequently, a majority of patients preferred the digital denture after experiencing both types. Within the limitations of the study, these results support the clinical viability of digital denture workflows, suggesting that modern CAD/CAM dentures are a satisfactory alternative to traditional dentures. Digital technology can be incorporated into complete denture fabrication without compromising (and potentially enhancing) patient satisfaction, though careful technique remains essential. Larger-scale studies are warranted to confirm these findings and further inform best practices in prosthodontic rehabilitation for edentulous patients.

## Figures and Tables

**Table 1:** Patient satisfaction questionnaire (PSQ) completed by the patients.

*Instructions*: Please rate your level of satisfaction with your complete dentures by selecting the most appropriate response for each statement.					
Item	1 Very Low	2 Low	3 Neutral	4 High	5 Very High
1. Comfort: How comfortable are your dentures when wearing them?	☐	☐	☐	☐	☐
2. Aesthetics: How happy are you with the way your dentures look?	☐	☐	☐	☐	☐
3. Ease of insertion and removal: How easy is it for you to put in and take out your dentures?	☐	☐	☐	☐	☐
4. Speech clarity: How well can you speak while wearing your dentures?	☐	☐	☐	☐	☐
5. Chewing efficiency: How well are you able to chew food with your dentures?	☐	☐	☐	☐	☐
6. Retention and stability: How well do your dentures stay in place during daily use?	☐	☐	☐	☐	☐
7. Sore spots and irritation: Have you experienced pain, sore spots, or irritation from your dentures?	☐	☐	☐	☐	☐
8. Need for adjustments: How often have you needed denture adjustments?	☐	☐	☐	☐	☐
9. Overall satisfaction: How satisfied are you overall with your complete dentures?	☐	☐	☐	☐	☐
10. Recommendation: Which denture would you recommend to other patients?	☐ Conventional ☐ Digital				
*Scoring and interpretation*:					
*Responses can be converted into numerical values (1–5) for statistical analysis*. *Higher scores indicate greater patient satisfaction*.					

**Table 2:** Baseline characteristics of the included participants.

Patient’s Age (Years) Mean (±SD)	Sex	Opposing Mandibular Arch
65.3 ± 8.7	Male 24 (60%)	Natural teeth— Complete denture 18 (45%) Removable partial denture 14 (35%)
	Female 16 (40%)	Fixed prosthetic restorations 8 (20%)

**Table 3:** Comparison of patient satisfaction (PSQ) for both types of complete maxillary dentures at T1 and T2.

Items from PSQ	C-CD (T1) Median (SD)	C-CD (T1) Median (SD)	p T1	C-CD (T2) Median (SD)	C-CD (T2) Median (SD)	p T2
**1. Comfort**	4 (0.3)	5 (0.4)	0.04 *	4 (0.6)	5 (0.2)	<0.01 *
**2. Aesthetics**	3 (0.4)	4 (0.3)	0.79	4 (0.5)	5 (0.3)	0.03 *
**3. Stability**	3 (0.6)	4 (0.5)	0.40	4 (0.6)	5 (0.4)	<0.01 *
**4. Speech**	4 (0.5)	4 (0.4)	0.08	4 (0.5)	5 (0.3)	<0.01 *
**5. Retention**	3 (0.6)	5 (0.5)	0.04 *	4 (0.7)	5 (0.4)	<0.01 *
**6. Mastication**	4 (0.7)	4 (0.6)	0.04 *	4 (0.6)	4 (0.5)	<0.01 *
**7. Need for adjustments**	4 (0.6)	5 (0.4)	0.02 *	3 (0.7)	5 (0.3)	<0.01 *
**8. Oral condition**	3 (0.5)	4 (0.3)	0.09	4 (0.6)	5 (0.4)	0.03 *
**9. Overall satisfaction**	4 (0.7)	4 (0.5)	0.06	4 (0.8)	5 (0.3)	<0.01 *

* Significant differences, *p* ≤ 0.05 (Wilcoxon signed-rank test for paired data)

**Table 4:** Internal consistency (consistency of patients between responses).

Set	Cronbach’s α *
**C-CD T_1_**	>0.9
**D-CD T_1_**	>0.9
**C-CD T_2_**	>0.9
**D-CD T_2_**	>0.9

* Cronbach’s alpha coefficient > 0.90.

## Data Availability

The data presented in this study are available on request from the corresponding author.
